# A Spatio-Temporal Analysis of the Health Situation in Poland Based on Functional Discriminant Coordinates

**DOI:** 10.3390/ijerph18031109

**Published:** 2021-01-27

**Authors:** Mirosław Krzyśko, Waldemar Wołyńki, Marcin Szymkowiak, Andrzej Wojtyła

**Affiliations:** 1 Interfaculty Institute of Mathematics and Statistics, Calisia University-Kalisz, 62-800 Kalisz, Poland; mkrzysko@amu.edu.pl; 2 Faculty of Mathematics and Computer Science, Adam Mickiewicz University, 61-614 Poznań, Poland; 3 Institute of Informatics and Quantitative Economics, Poznań University of Economics and Business, 61-875 Poznań, Poland; m.szymkowiak@ue.poznan.pl; 4 Statistical Office in Poznań, 60-624 Poznań, Poland; 5 Health Sciences Faculty, Calisia University-Kalisz, 62-800 Kalisz, Poland; a.wojtyla55@gmail.com

**Keywords:** health policy, health inequalities, healthcare access, spatial distribution of the health situation, cluster analysis, functional discriminant coordinates, multivariate functional coefficient of variation, spatio-temporal data

## Abstract

The aim of this study was to investigate if the provinces of Poland are homogeneous in terms of the observed spatio-temporal data characterizing the health situation of their inhabitants. The health situation is understood as a set of selected factors influencing inhabitants’ health and the healthcare system in their area of residence. So far, studies concerning the health situation of selected territorial units have been based on data relating to a specific year rather than longer periods. The task of assessing province homogeneity was carried out in two stages. In stage one, the original spatio-temporal data space (space of multivariate time series) was transformed into a functional discriminant coordinates space. The resulting functional discriminant coordinates are synthetic measures of the health situation of inhabitants of particular provinces. These measures contain complete information regarding 8 diagnostic variables examined over a period of 6 years. In the second stage, the Ward method, commonly used in cluster analysis, was applied in order to identify groups of homogeneous provinces in the space of functional discriminant coordinates. Sixteen provinces were divided into four clusters. The homogeneity of the clusters was confirmed by the multivariate functional coefficient of variation.

## 1. Introduction

Health is universally regarded as one of the most highly appreciated values. Good health is the main factor contributing to people’s well-being, which enhances their opportunities to participate in social life and to benefit from economic and employment growth. Better health is also consistently associated with greater life satisfaction (Ngamaba et al. [1]). A good health situation is instrumental in achieving good labor market outcomes. By reducing the individual’s capacity to work long hours, a deteriorated health status decreases their chance of getting employed and being productive at work and has a strong impact on the labor market situation (James et al. [2], OECD [3]).

For the purpose of this study, the term “health situation” is understood as inhabitants’ health described by the set of selected indicators and healthcare access in their area of residence. According to Penchansky and Thomas [4], healthcare access can be defined as a multi-faceted concept expressing the “degree of fit” between clients (patients) and the healthcare system according to five important dimensions: availability, accessibility, accommodation, affordability, and acceptability. In our article, we focus mainly on the first dimension, i.e., availability, which represents the “spatial” component of healthcare access. This choice is motivated by the fact that our understanding of availability is the same as that presented by Penchansky and Thomas [4], i.e., as the relationship between the number and type of existing services (and resources) and the number of patients and types of their needs. In other words, it represents the adequacy of the supply of physicians, dentists, and other providers; of facilities, such as clinics and hospitals; and of specialized programs and services, such as mental health and emergency care. The second dimension highlighted by Penchansky and Thomas [4], i.e., accessibility, also represents the “spatial” component of healthcare access and is understood as the relationship between the location of supply and the location of clients, while accounting for clients’ transportation resources and travel time, distance and cost. In our approach, however, we do not take this spatial component into account, mainly due to limited data availability. However, it should be emphasized that the literature on accessibility in the context of healthcare access is wide (see, for instance, Wang [5] and Neutens [6]). There is also a rich literature devoted to applications of the access concept to various health care services, e.g., regarding health care accessibility analyzed in connection with availability (Barbarisi et al. [7], Bruno et al. [8], Lu et al. [9], Okuyama et al. [10], Pu et al. [11]).

The importance attached to health by different organizations is reflected in the way health protection is implemented in public policies, particularly in health policy, which aims to have a positive influence on population health (De Leeuw et al. [12]). This policy can be understood as government decisions and plans of action to make progress towards achieving the goals of the health system: improved health status of the population, better financial risk protection, and better client satisfaction; or intermediate outcomes for health systems, which include: quality, access, and efficiency (Campos and Reich [13]). In many countries, the main objective of public health policy is to create the conditions for good and equitable health for the entire population and within specific groups and to eliminate avoidable health inequalities. It should also be underlined that decisions made in sectors outside of public health and health care, such as education, transportation, and criminal justice, strongly affect health and well-being (Pollack et al. [14]).

As mentioned above, the main objective of activities in the area of health policy is to improve the health of the population, and this improvement is nowadays understood in two ways (Łyszczarz [15]). First, in terms of improving the average health status, e.g., measured in terms of life expectancy or premature mortality. Second, increasing the importance attached to the issue of inequalities in health. The term ‘health inequality’ refers to differences in the health of individuals or specific subgroups; any measurable aspect of health that varies across individuals or according to socially relevant groupings can be called a health inequality (Boyle [16], Kawachi et al. [17], Arcaya et al. [18]). The aim of actions in the field of health policy is to reduce such inequalities. Large inequalities in health status exist across population groups, countries and specific regions within countries (Wojtyła-Buciora et al. [19], Wojtyła et al. [20]). These health inequalities are linked to many factors, including differential exposure to health risk factors and access to health care (Samet [21]). Inequalities in health are mainly manifested as differences in the health status between socioeconomic groups, but they can also described in terms of employment status, sex or geographic location (Crombie et al. [22]). The prospect of reducing inequalities seems to be increasingly important in contemporary research trends on the implementation of health policy in the world (see, e.g., Spinakis et al. [23]). It should also be emphasized that health inequalities have to be considered as a global problem, which not only affects populations of the poorest countries and regions but also those of the richest ones; persistent health inequalities are among the most serious and challenging health problems worldwide (Barreto [24]). How policies can reduce the main factors of health inequality and promote health equality will be a key challenge for public health in the future.

Monitoring population health and eliminating health inequalities are essential activities aimed at maintaining and improving public health. The main goals of health monitoring involve measuring the extent of health problems, their trends, and the degree of variation between different population groups, including spatial distribution, as well as identifying priority areas for public health (Pizot et al. [25]). Another objective of monitoring is to track the current health situation at the national and local level. This is especially important today, in the era of the Covid-19 pandemic, when up-to-date information is required at lower levels of spatial aggregation.

The unequal spatial distribution of resources, such as clinics, hospitals, nurses, pharmacies, or doctors, could make entire communities more vulnerable and less resilient to adverse health effects. That is why the health situation needs to be investigated by accounting for spatial differences to gain a deeper understanding of why and how some geographical areas experience different health than others (Ozdenerol [26]). Understanding the role played by location in shaping the geographic distribution of the health situation within countries is critical for informing appropriate public health policy regarding prevention and treatment (Casper et al. [27]).

There are numerous articles about the spatial variation in the health situation, health inequalities or health conditions at the local or national level (see, for instance, Gilliland et al. [28], Wang and Nie [29], Chen et al. [30]). In the case of Poland, various analyses have been conducted to investigate regional inequalities in the health status of the population (Wierzbicka [31], Bem et al. [32]).

Interestingly, all studies mentioned above were based on data for a specific year or, in some cases where comparative analysis was involved, for two years (e.g., Shi et al. [33], Hübelová et al. [34]). If the authors of these articles chose, say, *p* variables describing the health situation of a given territorial unit, then the obtained data were *p*-dimensional vectors or points in a *p*-dimensional Euclidean space.

This article presents a more general approach to investigating the health situation across territorial units, which is based on spatio-temporal data. This kind of data is more general than static vector data as it takes into account changes that happen over time. The statistical methodology involving the use of functional discriminant coordinates and cluster analysis is applied to available data for Poland. However, this approach can be used to investigate the health situation or other phenomena at lower levels of spatial aggregation in other countries. For this reason, its results may be useful for policy-makers in the field of public health. The data to measure the health situation in Poland come from the Local Data Bank (LDB). Several important variables related to the health situation observed at the level of districts (LAU—also called poviats) located within provinces (approximately equivalent to NUTS2 (regions) level and also called voivodships) (see Section 2) in the period 2013–2018 were taken into account in the analysis. More specifically, each district is described by 8 variables representing the situation over 6 years. The data for 380 districts were arranged in the form of a matrix with 6 rows and 8 columns, containing a total of 18,240 numerical values.

The main aim of this article is to determine whether Polish provinces are homogeneous in terms of spatio-temporal data characterizing their health situation. In order to answer this question, three multivariate statistical methods were used: multivariate functional discriminant coordinates analysis (MFDCA), functional cluster analysis (FCA), and the multivariate functional coefficient of variation (MFCV).

In the first step, spatio-temporal data were transformed into functional data by applying a continuous function of time *t* (see, e.g., Górecki and Krzyśko [35]). Functional data can be regarded as realizations of the random process X(t). Then, functional discriminant coordinates were constructed in the functional data space, and further calculations were performed in the functional discriminant coordinate space.

At this point, an important question arises: do the functional data recorded as continuous functions really exist and can these multivariate functions actually be derived? This question is critical because, in practice, values of an observed random process are always recorded in discrete moments in time, sparsely or densely distributed in the interval of variability over time. Thus, in this case, we encounter a time series or, in other words, a highly-dimensional vector of observations. However, there are numerous reasons why it is useful to model a time series as a continuous function (elements of a certain functional space); one of them is that functional data have many advantages in comparison to other representations of time series. In particular, the MFDCA derived in the present study has the following statistical advantages:

- Firstly, functional data are normally used to cope with the problem of missing observations, which is inevitable in many areas of applied research. Unfortunately, most methods concerning data analysis require complete time series. The removal of a time series with missing observations from a data set is one of popular solutions, but this can lead, and in most cases does lead, to serious data loss. Another possibility is to use one of the many methods of missing data prediction, but, in that case, the results will depend on the interpolation method. Contrary to these approaches, in the case of functional data, the problem of missing observations is resolved by expressing a given time series in the form of a continuous function set.
- Secondly, in the statistical development of MFDCA, the structure of observations is naturally retained when using functional data, i.e., the temporal link is maintained and the information regarding any measurement is taken into account. Consequently, results are assumed to be robust.
- Thirdly, moments of observation do not have to be equally spaced in a particular time series, which can be a major advantage in online applications.
- Fourthly, when using functional data, one avoids the problem of dimensionality. When the total number of time points in which observations are made exceeds the number of time series under analysis, most statistical methods do not provide satisfactory results because of misleading false estimates. In the case of functional data, this problem can be avoided because the time series are replaced by a set of continuous representative functions, which are independent of the time points in which observations are made.

The construction of functional discriminant coordinates is described in Górecki et al. [36], and their application to fruit data can be found in Hanusz et al. [37]. Two other proposals are: kernel discriminant coordinates (Krzyśko et al. [38]) and discriminant coordinates with the additional condition imposed on the covariance matrix (Krzyśko et al. [39]).

In the second step, cluster analysis was used to distinguish between groups of homogeneous provinces. Ward’s hierarchical clustering method was chosen as a commonly used technique in cluster analysis. Moreover, to determine whether obtained clusters are homogeneous, a functional multivariate coefficient of variation was applied.

The main value of this article, according to its authors, is the proposed statistical methodology. Despite the use of country-specific data for the purpose of spatial analysis of the health situation, the presented methods are universal and can be successfully applied to any territorial unit and spatio-temporal dynamic data connected to other phenomena (e.g., poverty or the labor market situation at lower levels of spatial aggregation).

This article is organized as follows. Section 2 contains a short description of data used to analyze differences in the health situation across Polish provinces. The section also provides a description of the administrative division of Poland and details of the procedure of data standardization, as well as their transformation into functional data. Section 3 presents the statistical methodology involving the use of functional discriminant coordinates, cluster analysis, and the functional multivariate coefficient of variation. How this approach was applied to real data describing the health situation in Poland is described in Section 4. Finally, concluding remarks and further steps to be taken in the future are provided in Section 5.

## 2. The Data

The original data set contains values of p=8 variables characterizing the health situation of the population (see Table 1). All variables come from the LDB, which is Poland’s largest database of information relating to the economy, society and the environment. Data and statistical indicators in the LDB describe entire country, as well as units representing three NUTS levels: macroregions (NUTS1), regions (NUTS2), and subregions (NUTS3).

Table 1 also contains information about variable type, with S denoting the so-called stimulant, where a higher value means a better situation (in terms of health), and D denoting the so-called destimulant, where lower values represent a better situation (Walesiak and Dudek [40]).

The variables were selected with a view to obtaining a relatively comprehensive description of the health situation of the population and taking into account their availability and completeness. The data cover the period 2013–2018, i.e., T=6 years and describe n=380 districts located within 16 provinces (see Table 2).

**Figure 1:**
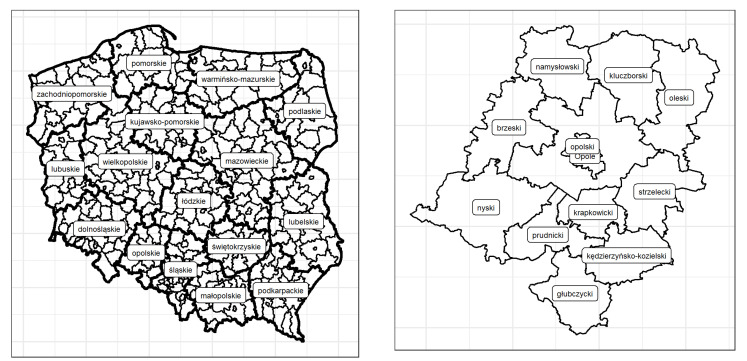
Administrative division of Poland—provinces and districts.

Provinces are essentially equivalent to NUTS2 units, while districts are the upper level of local administrative units, which are currently not part of the NUTS system. The NUTS classification (Nomenclature of territorial units for statistics) is a geographical standard used for a statistical division of the EU Member States economic territories into three regional levels of specified classes of the population. It was established in order to enable the collection, compilation, and dissemination of harmonized regional statistics in the European Union. More information about the administrative division of Poland can be found at https://stat.gov.pl/en/regional-statistics/classification-of-territorial-units/administrative-division-of-poland/. Figure 1 shows the administrative division of Poland into provinces and districts (the left panel) and the division of opolskie (as an example) into districts (the right panel).

The values of the selected variables, expressed in different measurement units and having different ranges of variation, were standardized using the method of zero unitization (see, for example, Jajuga and Walesiak [41]).

Subsequently, the unitized data were transformed into functional data using the least squares method (see, e.g., Górecki and Krzyśko [35]).

Now, let us assume that the *d*-th component of the Z process can be represented by a finite number of orthonormal basis functions $\left\{\varphi_{b}\right\}$:

$$Z_{d}\left(t\right)=\sum_{b=0}^{B_{d}}\alpha_{db}\varphi_{b}\left(t\right),\;t\in I,\;d=1,2,\ldots ,p,$$

where $\alpha_{db}$ are random variables such that $\mathrm{Var}(\alpha_{db})<\infty$ for d=1,2,…,p and $b=0,1,\ldots ,B_{d}$.

Let

$$\alpha ={\left(\alpha_{10},\ldots ,\alpha_{1B_{1}},\ldots ,\alpha_{p0},\ldots ,\alpha_{pB_{p}}\right)}^{\prime }$$

and

$$\Phi \left(t\right)=\left[\begin{array}{cccc} \varphi_{B_{1}}^{\prime }\left(t\right) & 0^{\prime } & \ldots & 0^{\prime }\\ 0^{\prime } & \varphi_{B_{2}}^{\prime }\left(t\right) & \ldots & 0^{\prime }\\ \ldots & \ldots & \ldots & \ldots \\ 0^{\prime } & 0^{\prime } & \ldots & \varphi_{B_{p}}^{\prime }\left(t\right) \end{array}\right],$$

where $\varphi_{B_{d}}={\left(\varphi_{0},\ldots ,\varphi_{B_{d}}\right)}^{\prime }$, d=1,…,p, $\alpha \in R^{K+p},\Phi \in R^{p\times (K+p)},K=B_{1}+\ldots +B_{p}$. Then,

(1) Z(t)=Φ(t)α,t∈I.

Individual years (time points) were assigned the following values: $t_{1}=0.5\;\left(2013\right),t_{2}=1.5\;\left(2014\right),\ldots ,t_{6}=5.5\;\left(2018\right)$. The ϕ functions are considered on the interval I=[0,T]=[0,6]. The Fourier base of the form

$$\phi_{0}\left(t\right)=1/\sqrt{T},\;\phi_{2k-1}\left(t\right)=\sqrt{2/T}\sin\left(2\pi kt/T\right),\;\phi_{2k}\left(t\right)=\sqrt{2/T}\cos\left(2\pi kt/T\right),$$

where t∈[0,T], k=1,2,…, was adopted as the orthonormal basis. Górecki and Krzyśko [35] showed that the Fourier base leads to a minimal number of terms in the expansion of a given function into a series, which is a desirable feature because expansion coefficients play the role of new variables in the functional approach. Given the small number of time points, for each of the 8 variables, the number of expansion terms was the same and equal to 5. Hence, $B_{1}=\ldots =B_{8}=4$, $K=B_{1}+\cdots +B_{8}=32$, K+p=40. Thus, $\alpha \in R^{40}$ and $\Phi \in R^{8\times 40}$.

**Figure 2:**
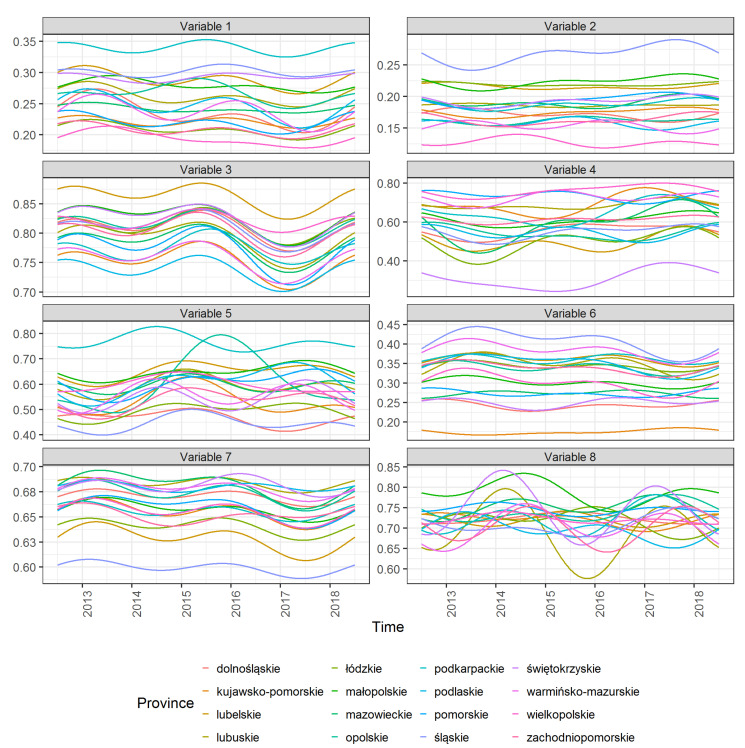
Average values of 8 variables calculated from functional data for districts included in each of the 16 provinces. **Note:** The ordinate axis shows the unitized values of a given variable.

Figure 2 shows the functional data (average values) for 8 variables and 16 provinces. One can see how the values of individual variables vary over time and between provinces.

## 3. Statistical Methodology

### 3.1. Functional Discriminant Coordinates

Our purpose is to construct a discriminant coordinate based on multivariate functional data, i.e., to construct

$$U=<u,Z>=\int_{I}u^{\prime }\left(t\right)Z\left(t\right)dt$$

such that their between-class variance is maximal compared with the within-class variance, where

u(t)=Φ(t)γ.

The construction of functional discriminant coordinates is described in Górecki et al. [36] and Hanusz et al. [37].

The construction of discriminant coordinates for the random process Z essentially consists in constructing classical discriminant coordinates for a random vector α because the discriminant component $U_{k}$ has the form $U_{k}=\gamma_{k}^{\prime }\alpha$, where α is the random vector in the representation Z(t)=Φ(t)α of the random process Z, and $\gamma_{k}$ is an eigenvector in the generalized eigenproblem $\left(B-\lambda_{k}W\right)\gamma_{k}=0$, where B and W are the between-class and within-class matrices, respectively.

**Remark** **1.** *The examination of the elements of the vector weight function for the original processes in each discriminant coordinate (elements of the vectors $u_{k}$) helps to interpret the principal axes of between-class variation.*

At a given time point *t*, the greater the absolute value of a component of the vector weight function, the greater the contribution in the structure of the given functional discriminant coordinate, from the process Z corresponding to that component. The total contribution of a particular original process $Z_{i}$ in the structure of a particular functional discriminant coordinate is equal to the area under the module weighting function corresponding to this process.

In practice, vector α is unknown and must be estimated based on the sample. Let $z_{i1},z_{i2},\ldots ,z_{in_{i}}$ be a sample belonging to the *i*-th class, where i=1,2,…,L. The function $z_{ij}$ has the form

$$z_{ij}\left(t\right)=\Phi \left(t\right)a_{ij},$$

where $a_{ij}={\left(a_{10}^{\left(ij\right)},\ldots ,a_{1K_{1}}^{\left(ij\right)},\ldots ,a_{p0}^{\left(ij\right)},\ldots ,a_{pK_{p}}^{\left(ij\right)}\right)}^{\prime }$, $i=1,2,\ldots ,L,j=1,2,\ldots ,n_{i}$.

Let

$${\bar{a}}_{i}=\frac{1}{n_{i}}\sum_{j=1}^{n_{i}}a_{ij},\;\bar{a}=\frac{1}{n}\sum_{i=1}^{L}n_{i}{\bar{a}}_{i},\;i=1,\ldots ,L,\;n=n_{1}+\cdots +n_{L}.$$

Then,

$$\hat{B}=\frac{1}{L-1}\sum_{i=1}^{L}n_{i}\left({\bar{a}}_{i}-\bar{a}\right){\left({\bar{a}}_{i}-\bar{a}\right)}^{\prime },$$

$$\hat{W}=\frac{1}{n-L}\sum_{i=1}^{L}\sum_{j=1}^{n_{i}}\left(a_{ij}-{\bar{a}}_{i}\right){\left(a_{ij}-{\bar{a}}_{i}\right)}^{\prime }.$$

Next, we find the non-zero eigenvalues ${\hat{\lambda }}_{1}^{🟉}\geq {\hat{\lambda }}_{2}^{🟉}\geq \ldots \geq {\hat{\lambda }}_{s}^{🟉}$ and the corresponding eigenvectors ${\hat{\gamma }}_{1},{\hat{\gamma }}_{2},\ldots ,{\hat{\gamma }}_{s}$ of the matrix ${\hat{W}}^{-1}\hat{B}$, where s=min(K+p,L−1). Hence,

$${\hat{u}}_{k}\left(t\right)=\Phi \left(t\right){\hat{\gamma }}_{k},$$

and the coefficients of the projection of the *j*-th realization $z_{ij}$ of the process Z belonging to the *i*-th class on the *k*-th functional discriminant coordinate are equal to:

$${\hat{U}}_{ijk}=<{\hat{u}}_{k},z_{ij}>={\hat{\gamma }}_{k}^{\prime }a_{ij},$$

for $i=1,2,\ldots ,L,j=1,2,\ldots ,n_{i},k=1,2,\ldots ,s$.

The plots of the pairs $\left({\hat{U}}_{ij1},{\hat{U}}_{ij2}\right)$ provide a visual representation of the relative position of groups in the two-dimensional space. Since the configuration obtained is deemed to be optimal in terms of the ability to discriminate between the groups, wide overlaps are to be considered as a sign of no or small differences between the groups involved.

### 3.2. Cluster Analysis

Provinces that are homogeneous in terms of the considered variables were identified using cluster analysis. More precisely, we applied Ward’s hierarchical clustering method (see, for example, Seber [42], Chapter 7; Mirkin [43]; Krzyśko et al. [44], Chapter 12). The clustering procedure is based on the Mahalanobis distance between the provinces.

Let ${\hat{U}}_{ij}={\left({\hat{U}}_{ij1},\ldots ,{\hat{U}}_{ijs}\right)}^{\prime }$. This distance is defined by the following formula:

$$d_{ij}^{2}={\left({\bar{U}}_{i}-{\bar{U}}_{j}\right)}^{\prime }S^{-1}\left({\bar{U}}_{i}-{\bar{U}}_{j}\right),$$

where

$${\bar{U}}_{i}=\frac{1}{n_{i}}\sum_{j=1}^{n_{i}}{\hat{U}}_{ij},\;i=1,2,\ldots ,L,$$

and

$$S=\frac{1}{n-L}\sum_{i=1}^{L}\sum_{j=1}^{n_{i}}\left({\hat{U}}_{ij}-{\bar{U}}_{i}\right){\left({\hat{U}}_{ij}-{\bar{U}}_{i}\right)}^{\prime },\;n=n_{1}+\ldots +n_{L}.$$

The Mahalanobis distance takes into account not only the difference between the mean vectors of two provinces; the difference is also weighted by the variances and covariances of the examined variables estimated for all provinces (the differentiation of districts around the mean provinces was taken into account).

### 3.3. Functional Multivariate Coefficient of Variation

Let $Z={\left(Z_{1},\ldots ,Z_{p}\right)}^{\prime }$, be a *p*-dimensional random process with mean function $\mu ={\left(\mu_{1},\ldots ,\mu_{p}\right)}^{\prime }\neq 0$. We assume that the process Z belongs to the Hilbert space $L_{2}^{p}\left(I\right)$ of *p*-dimensional vectors of square integrable functions on *I*.

The functional multivariate coefficient of variation (MFCV) for the random process Z is defined as follows (Krzyśko and Smaga [45])

$$\mathrm{MFCV}=\frac{\sqrt{\mathrm{Var}(<\mu_{*},Z>}}{∥\mu ∥},$$

where $\mu_{*}\left(t\right)=\mu \left(t\right)/∥\mu ∥$, t∈I.

If process Z has the form (1), then

$$\mathrm{MFCV}=\sqrt{\frac{a^{\prime }J_{\Phi }\Sigma_{\alpha }J_{\Phi }a}{{\left(a^{\prime }J_{\Phi }a\right)}^{2}}},$$

where $J_{\Phi }=\mathrm{diag}\left(J_{\phi_{1}},\ldots ,J_{\phi_{p}}\right)$, $J_{\phi_{k}}=\int_{I}\phi_{k}\left(t\right)\phi_{k}^{\prime }\left(t\right)dt$, and $\Sigma_{\alpha }=\mathrm{Cov}\left(\alpha \right)$ is the $B_{k}\times B_{k}$ cross product matrix corresponding to the basis ${\left\{\phi_{kl}\right\}}_{l=1}^{\infty }$, k=1,…,p. For the orthonormal basis, for instance the Fourier basis, the cross product matrix is equal to the identity matrix. Then (Albert and Zhang [46]),

$$\mathrm{MFCV}=\sqrt{\frac{a^{\prime }\Sigma_{\alpha }a}{{\left(a^{\prime }a\right)}^{2}}}.$$

## 4. Results

To construct functional discriminant coordinates, we calculated the estimates $a_{i}$ of the vectors $\alpha_{i}$, i=1,…,L.

The vectors $a_{i}$ were then used to construct the estimator $\hat{B}$ of the matrix of between-class variability and the estimator $\hat{W}$ of the matrix of within-class variability. Next, the non-zero eigenvalues of ${\hat{\lambda }}_{k}^{🟉}$ of the matrix ${\hat{W}}^{-1}\hat{B}$ and the corresponding eigenvectors ${\hat{\gamma }}_{k}$, k=1,…,15, were calculated.

Multivariate functional discriminant coordinates have the form:

$${\hat{U}}_{k}=<{\hat{u}}_{k},Z>,$$

where

$${\hat{u}}_{k}\left(t\right)=\Phi \left(t\right){\hat{\gamma }}_{k},\;k=1,\ldots ,s,$$

s=min(K+p,L−1)=min(40,15)=15,

are vectors of weight functions.

We treat the resulting multivariate functional discriminant coordinates as indicators (synthetic measures) of the health situation of inhabitants of Polish provinces. These indicators contain full information on the values of 8 diagnostic variables measured over 6 years. They are, therefore, composite indicators of the health situation.

These 15 composite indicators have a different power of differentiating between the provinces (these indicators have different variances (eigenvalues); see Table 3).

The first indicator is the strongest and the fifteenth is the least powerful. It is not possible to see the mutual position of provinces in the 15-dimensional space of these indicators, but it is possible in the space of the first two composite indicators that differentiate between the provinces most clearly.

It can be noticed that 44.1% of total variability is attributed to the first two multivariate functional coordinates.

The mean values of the 16 provinces in the system of the first two functional discriminant coordinates are presented in Table 4.

**Figure 3:**
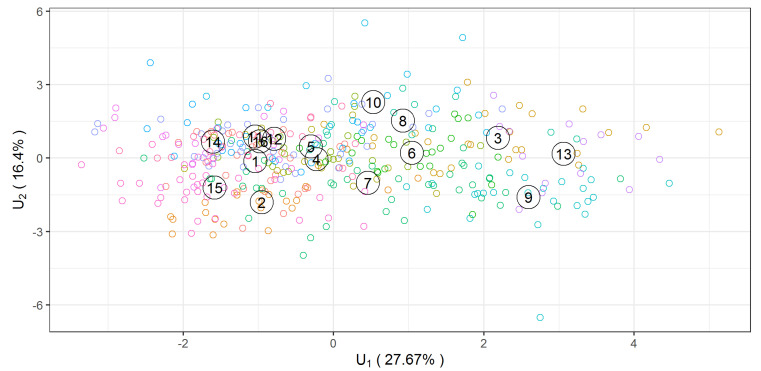
Plotted values of the first two functional discriminant coordinates.

The location of the 16 provinces in the system of the first two functional discriminant coordinates is shown in Figure 3.

**Figure 4:**
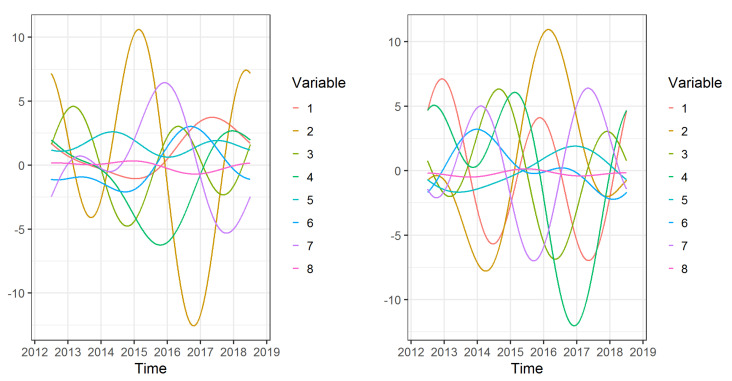
Weight functions for the first (left) and the second (right) functional discriminant coordinate.

The total contribution of the individual variables to the structure of the particular functional discriminant coordinates can be estimated using the area under the absolute value of the weight functions corresponding to a given variable. The graphs of the eight components of the vector weight function for the first and second functional discriminant coordinates are shown in Figure 4.

These contributions, for the first and second functional discriminant coordinates for 8 variables are also given in Table 5. Table 5 shows that the largest share in the construction of the first functional discriminant coordinate is played by variable No. 2 (Doctors per 10,000 population)—32.0%—and variable No. 7 (Number of doctor consultations per 10,000 population)—14.7%. On the other hand, variable No. 4 (Deaths of people due to cardiovascular disease per 100,000 population)—21.0%—and variable No. 2 (Doctors per 10,000 population)—20.2%—have the greatest share in the construction of the second functional discriminant coordinate. Values of coefficients of the vector weight functions are also presented in Table 5.

**Figure 5:**
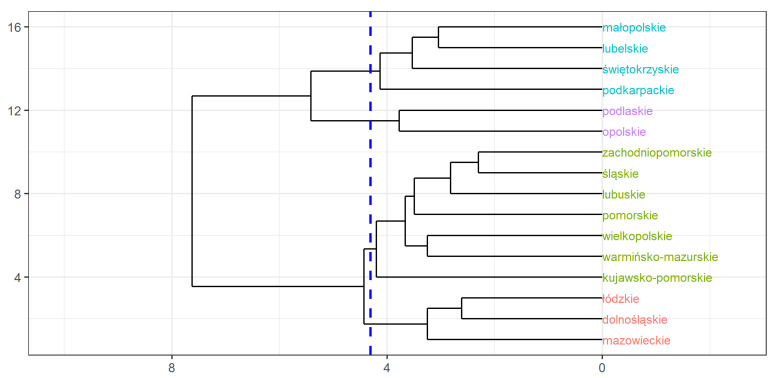
Dendrogram for 16 Polish provinces (the Ward method).

In the next step, cluster analysis was used to select groups of homogeneous provinces in a fifteen-dimensional space of functional discriminant coordinates. The Ward method was selected as a commonly used technique. The Mahalanobis distance was chosen as a measure of the distance between the mean vectors of individual provinces. The obtained dendrogram is presented in Figure 5.

**Figure 6:**
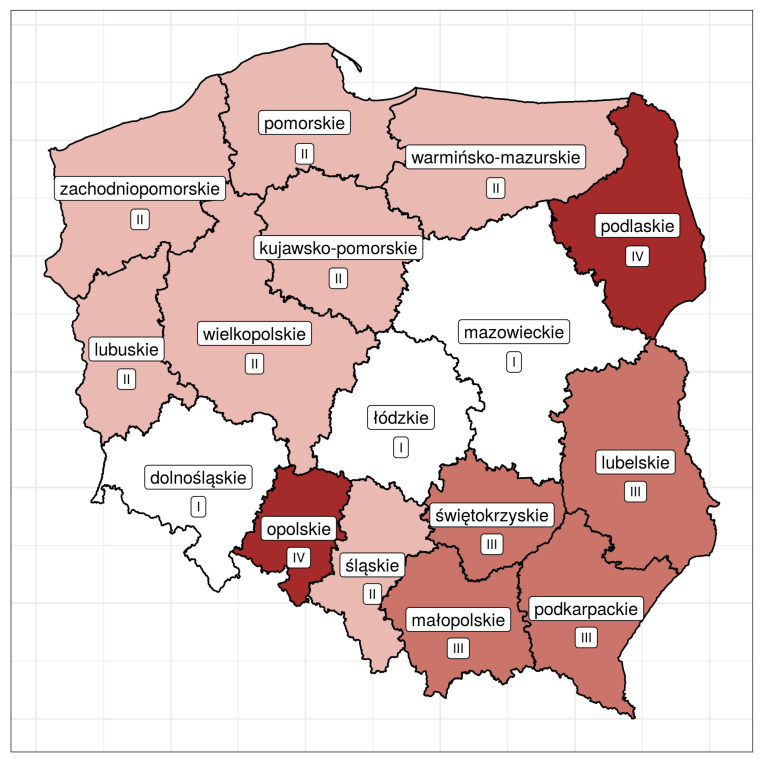
Spatial variation in the health situation across the provinces of Poland.

We obtained four homogeneous clusters. Which cluster individual provinces belong to is shown in Table 6 and in Figure 6 (spatial distribution).

Taking into account the spatial variation in the health situation of the provinces, it is possible to distinguish four spatial clusters. The first one, denoted as II, consists of six provinces located in the north-western part of Poland. At the opposite end of the country, there are four provinces that make up cluster III. Finally, in the approximate middle belt, one can see cluster I, consisting of three provinces. Cluster IV consists of two non-contiguous provinces (podlaskie and opolskie), located in different parts of Poland.

Figure 6 shows that Poland can be divided into two part: Western Poland (provinces belonging to clusters I and II) and Eastern Poland (provinces belonging to clusters III and IV). The health of inhabitants living in the provinces belonging to cluster I is the best, while that of people living in the provinces belonging to cluster IV is the worst. All the previous studies conducted in Poland show that Western Poland is better developed in socio-economic terms than Eastern Poland (see, for instance, Szymkowiak et al. [47], Marchetti et al. [48], Roszka [49]). Current research shows that this division is also valid as regards the health situation.

Decision-makers at national and local government levels should be advised to redirect more funds to improve the health situation of inhabitants of Easter Poland.

To verify that the obtained four clusters are really homogeneous, a multivariate functional coefficient of variation (MFCV) was calculated for all provinces together and for each cluster separately (see Table 7).

As can be seen, the coefficients of variation for individual clusters are lower than that for all indeed homogeneous.

## 5. Conclusions

The above statistical analysis provides evidence for the conclusion that the provinces are not homogeneous in terms of the selected variables characterizing the health situation of their inhabitants. The analysis consisted of multiple steps. Values of the selected variables, which are expressed in different measurement units and have different ranges of variation, were standardized using the method of zero unitization. Then, the unitized data were transformed into functional data in order to enable the construction of discriminant coordinates in the functional data space. The multivariate functional discriminant coordinates were treated as composite indicators (synthetic measures) of the health situation of inhabitants of Polish provinces. These indicators contain full information on the values of 8 diagnostic variables measured over 6 years.

In the next step, cluster analysis was applied to select groups of homogeneous provinces in the space of functional discriminant coordinates using the Ward method. The Mahalanobis distance was chosen as a measure of distance between the mean vectors of individual provinces. The homogeneity of the resulting four clusters was analyzed using a multivariate functional coefficient of variation, which was calculated for all provinces together and for each cluster separately. It turned out that the coefficients of variation for individual clusters are smaller than the corresponding value for combined provinces, which confirms that the clusters are indeed homogeneous.

The obtained clusters illustrate changes in the situation of the provinces (over a period of six analyzed years). In previous studies, data for each year are analyzed separately using classical statistical methods. However, one must not forget that one deals with spatio-temporal data that change over time.

The authors realize that the choice of diagnostic variables may be a weakness of this study. These particular diagnostic variables were selected with a view to obtaining relatively comprehensive description of the health situation of the population, given their availability and completeness. Therefore, the selection should be treated mainly as an illustration of the proposed statistical methodology for processing spatio-temporal data.

The statistical methods used for multivariate functional data were suggested earlier by the authors of this paper.

## Figures and Tables

**Table 1:** List of variables used in analysis.

Variable	Description	Type of Variable
1	Nurses and midwives per 10,000 population	S
2	Doctors per 10,000 population	S
3	Population per generally available pharmacy	D
4	Deaths of people due to cardiovascular disease per 100,000 population	D
5	Total deaths due to cancer per 100,000 population	D
6	Health out-patient departments per 10,000 population	S
7	Number of doctors consultations per 10,000 population	S
8	Infant deaths per 1000 live births	D

**Table 2:** The composition of Polish provinces.

Number	Province Name	Number of Districts
1	dolnośląskie	30
2	kujawsko-pomorskie	23
3	lubelskie	24
4	lubuskie	14
5	łódzkie	24
6	małopolskie	22
7	mazowieckie	42
8	opolskie	12
9	podkarpackie	25
10	podlaskie	17
11	pomorskie	20
12	śląskie	36
13	świętokrzyskie	14
14	warmińsko-mazurskie	21
15	wielkopolskie	35
16	zachodniopomorskie	21
	**Total**	**380**

**Table 3:** Eigenvalues and related statistics.

Number	Eigenvalue	% Total Variance	% Cumulative Variance
1	48.2682	27.6745	27.6745
2	28.6024	16.3991	44.0735
3	26.3461	15.1055	59.1790
4	15.1611	8.6926	67.8716
5	11.6214	6.6631	74.5347
6	9.4012	5.3901	79.9248
7	8.5436	4.8984	84.8232
8	5.6155	3.2197	88.0429
9	4.9859	2.8587	90.9016
10	4.8982	2.8083	93.7099
11	3.4169	1.9591	95.6690
12	2.7111	1.5544	97.2234
13	2.0385	1.1687	98.3921
14	1.4576	0.8357	99.2279
15	1.3467	0.7721	100.0000

**Table 4:** The mean values of the 16 provinces (first two).

Number	Variable 1	Variable 2
1	−1.0418	−0.1272
2	−0.9538	−1.8010
3	2.1856	0.8277
4	−0.2247	−0.0520
5	−0.3005	0.4795
6	1.0408	0.2084
7	0.4584	−1.0042
8	0.9223	1.5262
9	2.5934	−1.5963
10	0.5307	2.2876
11	−1.0424	0.8810
12	−0.7883	0.7896
13	3.0622	0.1767
14	−1.6026	0.6644
15	−1.5847	−1.2004
16	−0.9763	0.6872

**Table 5:** Values of coefficients of the vector weight functions.

**First functional discriminant coordinate**							
Variable	${\hat{\gamma }}_{10}$	${\hat{\gamma }}_{11}$	${\hat{\gamma }}_{12}$	${\hat{\gamma }}_{13}$	${\hat{\gamma }}_{14}$	Area	Area (%)
1	2.5407	−3.2389	2.0978	-0.3165	−0.9303	9.0039	8.2381
2	0.5792	7.8019	−0.7947	−7.3340	12.8083	34.9609	31.9876
3	0.2994	−0.5582	2.4586	6.3065	0.1062	14.4837	13.2519
4	−3.0143	0.3007	6.7926	−1.9114	−1.2470	15.1264	13.8399
5	3.8320	0.7027	0.1091	−0.9202	−0.7656	9.3864	8.5882
6	−0.1795	−3.5460	−0.4691	1.3195	−1.3298	8.7501	8.0059
7	0.9525	0.6116	−6.6553	4.7278	1.7190	16.0264	14.6634
8	−0.1802	0.6553	0.0063	−0.1974	0.3950	1.5574	1.4249
**Second functional discriminant coordinate**							
Variable	${\hat{\gamma }}_{20}$	${\hat{\gamma }}_{21}$	${\hat{\gamma }}_{22}$	${\hat{\gamma }}_{23}$	${\hat{\gamma }}_{24}$	Area	Area (%)
1	−0.8113	2.1021	1.9010	7.7922	6.7172	23.1732	17.3879
2	1.3009	−9.6903	−5.7988	6.8425	3.5944	26.8581	20.1529
3	0.2159	5.0384	0.8509	−7.6787	0.3243	18.8570	14.1493
4	−1.4582	10.6698	0.1764	−1.8355	8.9551	28.0178	21.0231
5	−0.0163	−2.8675	−0.7596	−0.2515	−0.4281	6.5687	4.9288
6	0.7540	2.9261	−1.3371	0.7459	−2.1031	7.4906	5.6206
7	1.2655	−0.1701	4.4019	−4.0271	−7.7531	20.7457	15.5665
8	−0.5597	−0.0570	−0.2735	−0.0484	0.3602	1.5604	1.1709

**Table 6:** Membership of provinces in the four clusters.

Number	Province	Cluster
1	dolnośląskie	I
2	kujawsko-pomorskie	II
3	lubelskie	III
4	lubuskie	II
5	łódzkie	I
6	małopolskie	III
7	mazowieckie	I
8	opolskie	IV
9	podkarpackie	III
10	podlaskie	IV
11	pomorskie	II
12	śląskie	II
13	świętokrzyskie	III
14	warmińsko-mazurskie	II
15	wielkopolskie	II
16	zachodniopomorskie	II

**Table 7:** Values of the multivariate functional coefficient of variation (MFCV).

Provinces	MFCV
All	0.3705
Cluster I	0.2975
Cluster II	0.2890
Cluster III	0.1728
Cluster IV	0.2047

## Data Availability

The data presented in this study are openly available in the Local Data Bank, which is Poland’s largest database of information relating to the economy, society and the environment.
